# The Philadelphia Society of Veterinary Medicine

**Published:** 1892-07

**Authors:** 


*The Philadelphia Society of Veterinary Medicine.*—The Philadelphia Society of Veterinary Medicine met at the College of Physicians, June 28th, 1892. This being a business meeting no papers were read. There were nineteen members present.

The Publication Committee reported. The committee were instructed by the society to have all the papers published if the expense did not exceed $125. The treasurer reported expenses for the year to be $60, and that the assessment, $2 per member, made in February, had been paid except by four members. It was moved that another assessment of $2 per member be made to meet expenses for the next six months, which was carried, all the members present paying, leaving the treasury in good condition for next year.

Election of officers—As the vice-president follows the president, Dr. Harger, being vice-president was made president, Dr. T. B. Rodgers, vice president ; Dr. Conrow, secretary ; Dr. Tagg, recording secretary ; Dr. Zuill, treasurer ; Dr. Felton, librarian ; executive committee, Drs. Ridge, Eaves, Fennimore. The executive committee acted upon five applications for membership. After a short address by the president the meeting adjourned to meet in September.

PRESIDENT'S ADDRESS.

I can well recollect while at college the desire to attend meetings of the different associations, but we never received an invitation. The association was composed of the select few. We could imagine what scientific papers and deep discussions were going on behind the closed door, and longed for the time when we also should gain that eminence and be accepted as members.

We can well remember seeing the members coming away from the meeting, still in deep argument as regards some of the points advanced.

Then, also, we would read later in the journals the paper as delivered, and wonder when we should ever gain enough knowledge to write such an essay. Then, as soon as we graduated we were accepted. Did we miss any meeting. Oh, no ! the novelty was too great to miss meetings. But were our expectations filled? No. After attending in one year ten meetings and only hearing four papers read, and on some nights no meeting, we soon began to think that we, too, might as well miss once in a while.

This soon left the association to be run by a few, with impulses by the addition of new members, who soon tired, especially when they were called upon to prepare a paper.

Now, what are we to do to improve this defect ? Are we to sit down and let others do all the work ? Then, if the meetings are not interesting, say : Oh, I told you so ; we cannot have as successful meetings as in the medical profession, because there are not enough of us.

Now, gentlemen, the Pennsylvania Association has seventy-nine members and will soon have one hundred ; our society thirty members, yet we have had difficulty in procuring papers for the last two meetings. Now, gentlemen, when an association with a membership like this one, has difficulty, we can readily see what trouble we might expect an association to have located in other parts of the State. We have certainly much the advantage over other associations, and it is our duty to give to the profession our experience. The journals are wanting it to give to their readers.

Now, because we are located favorably close to one of the best veterinary colleges in the United States, and among people who lean kindly to our profession, and the best talent is with us, it behooves us to be striving to make our society the best. How can we do this ?

We must keep an accurate record of our cases, then analyze them and give the results to the association. By keeping a close record of minute points by our expert practitioners, we will gain points that probably might go unobserved for a long while. These little things reported at our meetings make them interesting. Another way is to attend regularly. We always like to attend meetings where everybody else is present. Another point I want to bring before your notice to-night is, please do not forget that an association cannot be run without money. Of all the associations I belong to, that seems to be the chief trouble, so many of the members are delinquent.

Now, if you cannot attend by some circumstance you cannot control, please do not forget to pay your dues ; send it in by some member that you know attends ; do not wait until you can make it convenient to attend, for this cramps our finances and puts a burden on a few.

Now, I would say to those present who do not belong, to join our society, help bear our expenses, even if you *are* unable to attend. We intend to make this society one of the best, not only for its members, but by its publications, to be praised by the profession at large. Our membership is now composed of twenty-seven active members, three corresponding, and two applicants. The active members are : S. J. J. Harger, V. M. D., Wm. L. Zuill, M. D., D. V. S., Howard Felton, V. M. D., G. R. Hartman, V. M. D., J. J. Maher. V. M. D., Chas. Williams, V. M. D., W. H. Ridge, V. M. D., Chalkley Magill, V. M. D., Robt. Formad, V. M. D., E. Muir, V. M. D., E. H. Landes, V. M. D., M. E. Conrad, V.M.D., A. E. Conrow, M. D., V. M. D., H. D. Entriken, V. M. D., Elwood Bunting, V. M. D., B. F. Seuseman, V. M. D., Geonard Pearson, V. M. D., Wm. Birch, V. S., H. P. Eaves, V. M. D., J. P. Zuill, V. M. D., A. F. Schreiber, V. M. D., J. C. Bartholomew, V. M. D., Wm. Tagg, V. M. D., N. A. Cohen, M. D., V. D. M., Ph. G., H. Millar, V. M. D., H. D. Fennimore, D. V. S., T. B. Rodgers, D. V. S. Corresponding members are : Samuel G. Dixon, M. D., James C. McNeil, V. M. D., Simon Brimhall, V. M. D.

Now, gentlemen, I wish to thank you for the honor of being your first president and also for your kind assistance. I am to be followed by Dr. Harger, whose ability to further the interests of the society can be depended upon. I also thank our secretary for the able work he has performed. We all know what difficulties the first secretary labors under.

